# Prevalence, Clinical Severity, and Seasonality of Adenovirus 40/41, Astrovirus, Sapovirus, and Rotavirus Among Young Children With Moderate-to-Severe Diarrhea: Results From the Vaccine Impact on Diarrhea in Africa (VIDA) Study

**DOI:** 10.1093/cid/ciad060

**Published:** 2023-04-19

**Authors:** Adama Mamby Keita, Sanogo Doh, Samba O Sow, Helen Powell, Richard Omore, M Jahangir Hossain, Billy Ogwel, John B Ochieng, Joquina Chiquita M Jones, Syed M A Zaman, Alex O Awuor, Jane Juma, Dilruba Nasrin, Jie Liu, Awa Traoré, Uma Onwuchekwa, Henry Badji, Golam Sarwar, Martin Antonio, Eric R Houpt, Sharon M Tennant, Irene N Kasumba, Leslie P Jamka, Anna Roose, James A Platts-Mills, Jennifer R Verani, Jacqueline E Tate, Umesh D Parashar, Kathleen M Neuzil, Karen L Kotloff

**Affiliations:** Centre pour le Développement des Vaccins du Mali (CVD-Mali), Bamako, Mali; Centre pour le Développement des Vaccins du Mali (CVD-Mali), Bamako, Mali; Centre pour le Développement des Vaccins du Mali (CVD-Mali), Bamako, Mali; Center for Vaccine Development and Global Health, University of Maryland School of Medicine, Baltimore, Maryland, USA; Department of Pediatrics, University of Maryland School of Medicine, Baltimore, Maryland, USA; Kenya Medical Research Institute, Center for Global Health Research (KEMRI-CGHR), Kisumu, Kenya; Medical Research Council Unit The Gambia at the London School of Hygiene & Tropical Medicine, Banjul, The Gambia; Kenya Medical Research Institute, Center for Global Health Research (KEMRI-CGHR), Kisumu, Kenya; Kenya Medical Research Institute, Center for Global Health Research (KEMRI-CGHR), Kisumu, Kenya; Medical Research Council Unit The Gambia at the London School of Hygiene & Tropical Medicine, Banjul, The Gambia; Medical Research Council Unit The Gambia at the London School of Hygiene & Tropical Medicine, Banjul, The Gambia; Kenya Medical Research Institute, Center for Global Health Research (KEMRI-CGHR), Kisumu, Kenya; Kenya Medical Research Institute, Center for Global Health Research (KEMRI-CGHR), Kisumu, Kenya; Center for Vaccine Development and Global Health, University of Maryland School of Medicine, Baltimore, Maryland, USA; Department of Medicine, University of Maryland School of Medicine, Baltimore, Maryland, USA; Division of Infectious Diseases and International Health, Department of Medicine, University of Virginia, Charlottesville, Virginia, USA; Centre pour le Développement des Vaccins du Mali (CVD-Mali), Bamako, Mali; Centre pour le Développement des Vaccins du Mali (CVD-Mali), Bamako, Mali; Medical Research Council Unit The Gambia at the London School of Hygiene & Tropical Medicine, Banjul, The Gambia; Medical Research Council Unit The Gambia at the London School of Hygiene & Tropical Medicine, Banjul, The Gambia; Medical Research Council Unit The Gambia at the London School of Hygiene & Tropical Medicine, Banjul, The Gambia; Division of Infectious Diseases and International Health, Department of Medicine, University of Virginia, Charlottesville, Virginia, USA; Center for Vaccine Development and Global Health, University of Maryland School of Medicine, Baltimore, Maryland, USA; Department of Medicine, University of Maryland School of Medicine, Baltimore, Maryland, USA; Center for Vaccine Development and Global Health, University of Maryland School of Medicine, Baltimore, Maryland, USA; Department of Medicine, University of Maryland School of Medicine, Baltimore, Maryland, USA; Center for Vaccine Development and Global Health, University of Maryland School of Medicine, Baltimore, Maryland, USA; Department of Medicine, University of Maryland School of Medicine, Baltimore, Maryland, USA; Center for Vaccine Development and Global Health, University of Maryland School of Medicine, Baltimore, Maryland, USA; Department of Pediatrics, University of Maryland School of Medicine, Baltimore, Maryland, USA; Division of Infectious Diseases and International Health, Department of Medicine, University of Virginia, Charlottesville, Virginia, USA; Division of Global Health Protection, US Centers for Disease Control and Prevention, Nairobi, Kenya; Division of Viral Diseases, US Centers for Disease Control and Prevention, Atlanta, Georgia, USA; Division of Viral Diseases, US Centers for Disease Control and Prevention, Atlanta, Georgia, USA; Center for Vaccine Development and Global Health, University of Maryland School of Medicine, Baltimore, Maryland, USA; Department of Medicine, University of Maryland School of Medicine, Baltimore, Maryland, USA; Center for Vaccine Development and Global Health, University of Maryland School of Medicine, Baltimore, Maryland, USA; Department of Pediatrics, University of Maryland School of Medicine, Baltimore, Maryland, USA; Department of Medicine, University of Maryland School of Medicine, Baltimore, Maryland, USA

**Keywords:** diarrhea, adenovirus 40/41, astrovirus, sapovirus, rotavirus

## Abstract

**Background:**

While rotavirus causes severe diarrheal disease in children aged <5 years, data on other viral causes in sub-Saharan Africa are limited.

**Methods:**

In the Vaccine Impact on Diarrhea in Africa study (2015–2018), we analyzed stool from children aged 0–59 months with moderate-to-severe diarrhea (MSD) and without diarrhea (controls) in Kenya, Mali, and The Gambia using quantitative polymerase chain reaction. We derived the attributable fraction (AFe) based on the association between MSD and the pathogen, accounting for other pathogens, site, and age. A pathogen was attributable if the AFe was ≥0.5.

The severity of attributable MSD was defined by a modified Vesikari score (mVS). Monthly cases were plotted against temperature and rainfall to assess seasonality.

**Results:**

Among 4840 MSD cases, proportions attributed to rotavirus, adenovirus 40/41, astrovirus, and sapovirus were 12.6%, 2.7%, 2.9%, and 1.9%, respectively. Attributable rotavirus, adenovirus 40/41, and astrovirus MSD cases occurred at all sites, with mVS of 11, 10, and 7, respectively. MSD cases attributable to sapovirus occurred in Kenya, with mVS of 9. Astrovirus and adenovirus 40/41 peaked during the rainy season in The Gambia, while rotavirus peaked during the dry season in Mali and The Gambia.

**Conclusions:**

In sub-Saharan Africa, rotavirus was the most common cause of MSD; adenovirus 40/41, astrovirus, and sapovirus contributed to a lesser extent among children aged <5 years. Rotavirus- and adenovirus 40/41-attributable MSD were most severe. Seasonality varied by pathogen and location. Efforts to increase the coverage of rotavirus vaccines and to improve prevention and treatment for childhood diarrhea should continue.

## Teaser

Among children <5 years, rotavirus, adenovirus 40/41, and astrovirus cause moderate to severe diarrhea (MSD) in Kenya, Mali, and The Gambia, and sapovirus causes MSD in Kenya. Rotavirus and adenovirus 40/41 MSD have the highest severity.

Globally, diarrheal disease continues to negatively impact children aged <5 years, causing an estimated 500 000 deaths annually [1, 2]. Low-income countries are disproportionately affected, with 90% of diarrheal deaths occurring in sub-Saharan Africa and south Asia [3]. Prior to widespread vaccination, rotavirus was the leading cause of moderate-to-severe diarrhea (MSD) in children aged <5 years [4–7]. Other viruses associated with childhood diarrhea include norovirus, enteric adenovirus (in particular, serotypes 40 and 41), sapovirus, and astrovirus [8–11]. Previous research suggests that the prevalence of viruses as etiologic agents of pediatric diarrhea varies by geography, the severity of the diarrheal disease syndrome under study, and the level of sociodemographic development [12, 13].

Following successful clinical trials of rotavirus vaccines in low-resource countries and a World Health Organization (WHO) recommendation for routine use in all countries, rotavirus vaccines were introduced into national immunization programs in more than 110 countries globally between 2008 and 2016 [14–16]. An estimated 40% global reduction in rotavirus prevalence ensued and was associated with a decrease in rotavirus-associated diarrhea, hospitalizations, and deaths in children aged <5 years [17]. Despite these declines, children in Africa continue to die from diarrhea [18]. It is therefore critical to maintain an updated knowledge base of the etiologic agents associated with the most clinically relevant illnesses.

In addition to the positive health impact of rotavirus vaccine introduction, we anticipate a shift in predominant etiologies of MSD. Evidence from middle- and high-income countries post-rotavirus vaccine introduction indicates that viral agents, in particular, the caliciviruses (norovirus and sapovirus), have assumed the lead as the major causes of diarrheal hospitalizations and outpatient visits in children [19–23]. However, rigorous studies following rotavirus vaccine introduction are sparse in sub-Saharan Africa, where the need for relevant and impactful interventions is great. Such studies should test for an array of important pathogens using sensitive molecular microbiological diagnostics and study designs that account for asymptomatic infections to enable estimates of the proportion of pathogen detections that are attributable to the diarrheal syndromes associated with high morbidity and mortality [8, 24, 25].

The Global Enteric Multicenter Study (GEMS and GEMS-1A, 2007–2012) was a population-based, case-control study of the incidence, etiology, and adverse clinical consequence of medically attended diarrhea among children aged 0–59 months across 7 sites in Africa and Asia [4, 12]. Conducted prior to rotavirus vaccine introduction at any site, GEMS found that rotavirus, *Cryptosporidium*, *Shigella*, and heat-stable toxin-producing enterotoxigenic *Escherichia coli* had the highest attributable incidence among infants aged 0–11 months across a range of severity designated MSD [4] and less severe diarrhea (LSD) [8,12]. Enteric adenovirus 40/41 (using immunoassay) had the fifth highest attributable incidence in infants and the eighth highest in children aged 12–23 months [4]. Reanalysis of the GEMS data using the quantitative polymerase chain reaction (qPCR) assay resulted in a 5-fold increase in the attributable incidence of adenovirus 40/41, placing it as the second and third most common pathogen in these respective age groups [26]. Other viruses were less common.

The Vaccine Impact on Diarrhea in Africa (VIDA) study (2015–2018), a similarly designed follow-on to GEMS, reestimated the pathogen-specific burden associated with MSD after rotavirus vaccine introduction using qPCR methods in 3 of the GEMS African sites: The Gambia, Kenya, and Mali. The objectives of the research presented here were to describe the prevalence of episodes attributable to adenovirus 40/41, astrovirus, sapovirus, and rotavirus and the clinical characteristics of these cases; to examine the severity of attributable cases of adenovirus 40/41, astrovirus, and sapovirus compared with rotavirus; and to describe the seasonality of these viruses. The compelling need to better define the global burden of norovirus in low-income settings [27] is the subject of a separate analysis [28].

## METHODS

### Study Setting and Participants

VIDA is a prospective case-control study designed to assess etiology, incidence, and adverse clinical consequences of MSD among young children at 3 GEMS sites in sub-Saharan Africa (Kenya, Mali, and The Gambia). Participants were recruited at each site from a censused population using an ongoing demographic surveillance system (DSS). Cases were enrolled from sentinel health centers (SHCs) where DSS children sought care for diarrhea. MSD cases were defined as children aged 0–59 months who passed at least 3 abnormally loose stools within the past 24 hours, whose diarrhea started within the last 7 days, and who met at least 1 of the following criteria for MSD: sunken eyes, loss of skin turgor, dysentery, intravenous (IV) rehydration required, or hospitalization.

### Enrollment and Data Collection

All children from the DSS with diarrhea who presented to a SHC were screened for MSD according to published methods [29]. In brief, the sites aimed to enroll the first 8–9 MSD cases in each of 3 age strata (0–11 months, 12–23 months, 24–59 months) per fortnight for 36 consecutive months. For each case, 1–3 diarrhea-free control children were enrolled within 14 days of the case enrollment. Eligible controls were randomly selected from the DSS database, matched to the index case by age, sex, and residence [30].

At enrollment, the primary caregiver of each case and control underwent a standardized interview that comprised questions related to demographic, clinical, and epidemiologic features. A brief physical examination was performed that included anthropometric measurements. Caregivers were trained to use a simple memory aid card to record the occurrence of diarrhea each day for the next 14 days. Approximately 60 days after enrollment (targeted range, 50–90), cases and controls were visited at their homes to determine vital status, anthropometric measures, and interim clinical events and to review and collect the memory aid card.

For analyses of potential viral pathogens, a fresh, whole stool sample was obtained from each case within 12 hours of registration at a SHC and from each control at home; collection, transport, and processing were performed according to standardized methods [29]. For conventional analyses, samples were aliquoted into sterile tubes and stored at −80°C. Reverse-transcriptase PCR (RT-PCR) assays were used to analyze for astrovirus and sapovirus, and the ProSpecT Adenovirus Microplate (Oxoid, Basingstoke, UK) and the ProSpecT Rotavirus (Oxoid, Basingstoke, UK) commercial immunoassay kits were used to analyze for adenovirus and rotavirus, respectively. Samples positive for adenovirus common antigen were further tested using the Premier Adenoclone kit (Meridian Bioscience, Cincinnati, OH) to identify serotype 40/41.

Stool samples from all cases and their first matched control were also analyzed for viral pathogens using qPCR and TaqMan Array Card (TAC; Life Technologies, VA), according to Liu et al [26]. Standard curves were generated to convert the cycle threshold (Ct) to target copy numbers [31]. The quantity of pathogen was defined as log_10_-copy numbers per gram of stool. Pathogen positivity was defined as a Ct value <35, a standard cutoff [32]. Laboratory methods for viral pathogens are detailed elsewhere [29, 33, 34].

Monthly estimates of temperature and rainfall were obtained for each site. In The Gambia and Kenya, data came from government sources (The Gambia annual climate report and the Kenya Meteorological Department) with monitoring stations at major airports. Data for Mali came from Climate-Data.org [35], which provides monthly modeled estimates based on data from 1982–2012.

### Statistical Methods

#### Derivation of Attributable Cases

The episode-specific attributable fraction (AFe) was estimated for each child and pathogen using a pathogen-specific conditional logistic regression model [30] based on the TAC qPCR results that include the pathogen, an interaction between pathogen and site and between pathogen and age, as well as all other pathogens that occurred in at least 2% of cases and controls. Using the adjusted model coefficients, we estimated the episode-specific odds of an individual being a case (odds ratio [Or]) for each pathogen detected in their stool sample. An individual's odds were converted to an AFe based on 1 – 1/ORe. An MSD episode was considered attributable to an individual pathogen if the pathogen-specific AFe was ≥0.5 [36, 37].

#### Attributable Cases and Clinical Severity

We reported positivity for adenovirus 40/41, astrovirus, sapovirus, and rotavirus among cases and controls as defined by conventional methods (immunoassay and RT-RCR), qPCR positivity (Ct <35), and attributable MSD (AFe >0.5) using qPCR. We further reported the MSD cases that were attributed to 1 of these 4 viral pathogens by site, age, and sex. To compare the severity of clinical presentation of adenovirus 40/41-, astrovirus-, and sapovirus-attributed MSD with rotavirus-attributed MSD, we used a 20-point modified Vesikari score (mVS) [37], which contained all components of the original Vesikari score with minor modifications [7, 38] (Supplementary Table 1). Subgroup analyses by age and site were performed. The mVS made it possible to collect all data at enrollment except for duration of diarrhea, which was collected using the memory aid card. We categorized dehydration according to the WHO standard of none, some, and severe and the corresponding treatment guidelines [39]; a child who received IV fluids was considered more severe and counted as hospitalized, even if the child was not hospitalized [37]. The *χ*^2^ test (or the Fisher exact test) was used to compare categorical outcomes, while a Wilcoxon rank sum test was used for quantitative variables. Statistical significance was defined as a *P* value <.05. To ensure independent groups for statistical analyses, MSD episodes attributable to more than 1 of adenovirus 40/41, astrovirus, sapovirus, or rotavirus were excluded; if an episode was attributable to 1 of these viruses plus another putative pathogen, it was included.

#### Seasonality

To assess seasonality of adenovirus 40/41, astrovirus, sapovirus, and rotavirus, we calculated the total weighted number of pathogen-positive (Ct <35) MSD cases for each calendar month and summed over study years. A site- and age-specific weight of children who presented with MSD at SHCs divided by the number of cases enrolled was used to adjust for the cap on number of enrolled children. These monthly estimates were plotted alongside monthly maximum temperature (averaged over study years) and months when total rainfall (averaged over study years) exceeded mean rainfall for the study period.

All analyses were performed using R version 4.1.0.

## RESULTS

In VIDA, 4840 cases and 6213 controls were enrolled. All stool samples were analyzed using conventional methods, and 4836 cases and their first matched control were analyzed using qPCR. Less than 1% of tested samples did not produce a definitive result (Table 1).

**Table 1.** Prevalence of Viral Pathogen by Diagnostic Method for Cases and Controls and Percent of Cases Attributed to Each Virus for Cases Only During the Vaccine Impact on Diarrhea in Africa Study: 2015–2018

	Adenovirus 40/41		Astrovirus		Sapovirus		Rotavirus	
Diagnostic Method	Cases	Controls	Cases	Controls	Cases	Controls	Cases	Controls
RT-PCR/Immunoassay ^a^	4836	6209	4838	6209	4838	6209	4839	6213
ȃPositive, n (%)^b^	110 (2.3)	25 (0.4)	108 (2.2)	70 (1.1)	150 (3.1)	205 (3.3)	476 (9.8)	49 (0.8)
qPCR^a^	4708	4719	4803	4771	4806	4778	4806	4775
ȃPositive (cycle threshold <35), n^b^ (%)	584 (12.4)	541 (11.5)	304 (6.3)	211 (4.4)	668 (13.9)	531 (11.1)	644 (13.4)	208 (4.4)
ȃAttributable fraction (0.5), n (%)	127 (2.7)	…	141 (2.9)	…	90 (1.9)	…	609 (12.6)	…

Abbreviations: qPCR, quantitative polymerase chain reaction using the TaqMan Array Card; RT-PCR, reverse-transcription polymerase chain reaction.

Tested with definitive result.

Denominator is the number tested using RT-PCR/Enzyme immunoassay with definitive result.

### Prevalence

Compared with the “conventional” RT-PCR assay (sapovirus, astrovirus) or enzyme immunoassay (rotavirus, adenovirus 40/41), qPCR had higher positivity in both cases and controls for all 4 viruses (Table 1). The frequency of qPCR positivity among cases was 12.4%, 6.3%, 13.9%, and 13.4% for adenovirus 40/41, astrovirus, sapovirus, and rotavirus, respectively. When the prevalence of each virus in controls was taken into account by calculating the AFe, rotavirus accounted for 12.6% of MSD cases, while adenovirus 40/41, astrovirus, and sapovirus accounted for 2.7%, 2.9%, and 1.9% of MSD cases, respectively. Among the 910 total infections attributable to any of the 4 viruses, 20 (2.1%) were coinfections.

Episodes attributable to adenovirus 40/41, astrovirus, and rotavirus occurred in all age groups and at all sites. Only at the Kenya site were MSD cases attributable to sapovirus, with cases occurring across all age groups (Table 2). The majority of adenovirus 40/41-attributable MSD cases occurred in infants (0–11 months, 56.7%), while the majority of attributable astrovirus MSD cases occurred in toddlers (12–23 months, 59.6%). Attributable rotavirus MSD was notably present in both the infant and toddler groups (0–11 months, 42.5% and 12–23 months, 38.6%). Similar distributions were seen in males and females.

**Table 2.** Distribution of Attributable Moderate-to-Severe Diarrhea Cases by Study Site, Age Group, and Sex in the Vaccine Impact on Diarrhea in Africa Study

Subcategory		Adenovirus 40/41	Astrovirus	Sapovirus	Rotavirus
		n = 127	n = 141	n = 90	n = 609
Site	The Gambia	48 (37.8%)	50 (35.5%)	0	233 (38.3%)
	Mali	29 (22.8%)	48 (34.0%)	0	189 (31.0%)
	Kenya	50 (39.4%)	43 (30.5%)	90 (100.0%)	187 (30.7%)
Age, mo	0–11	72 (56.7%)	36 (25.5%)	24 (26.7%)	259 (42.5%)
	12–23	43 (33.9%)	84 (59.6%)	38 (42.2%)	235 (38.6%)
	24–59	12 (9.4%)	21 (14.9%)	28 (31.1%)	115 (18.9%)
	Median (Q1–Q3)	11 (8–15.5)	15 (11–20)	16.5 (11–25.75)	13 (9–20)
Sex	Male	64 (50.4%)	75 (53.2%)	52 (57.8%)	324 (53.2%)
	Female	63 (49.6%)	66 (46.8%)	38 (42.2%)	285 (46.8%)

### Clinical Severity

Based on the median (Q1–Q3) mVS, the severity of MSD caused by rotavirus was greatest (median mVS, 11 [8–12]), followed by adenovirus 40/41 (median mVS, 10 [7–11]), sapovirus (median mVS, 9 [7–11]), and astrovirus (median mVS, 7 [6–10]; Table 3). All mVS comparisons to rotavirus were statistically significant. When analyses were restricted to the youngest age group (0–11 months), the pattern of severity followed the same order: rotavirus mVS, 11 [8–12]; adenovirus mVS, 10.5 [7–12]; sapovirus mVS, 9 [8.5, 10]; and astrovirus mVS, 7 [6–10] (Supplementary Table 2).

**Table 3.** Clinical Severity of Adenovirus 40/41-, Astrovirus-, and Sapovirus-Attributable Cases in the Vaccine Impact on Diarrhea in Africa Study and Comparison With Rotavirus

Subcategory							*P* Values			
		Adenovirus 40/41 (n = 121)^a^	Astrovirus (n = 123)^a^	Sapovirus (n = 82) ^a^	Rotavirus		Adenovirus 40/41 vs Rotavirus	Astrovirus vs Rotavirus	Sapovirus vs Rotavirus	
					All (*n* = 584)^a^	Kenya (*n* = 180)^a^			All	Kenya
Modified Vesikari score	Mild	24 (19.8%)	45 (36.6%)	19 (23.2%)	78 (13.4%)	10 (5.6%)				
	Moderate	45 (37.2%)	54 (43.9%)	40 (48.8%)	202 (34.6%)	57 (31.7%)	.097	**<.001**	**<**.**001**	**<**.**001**
	Severe	52 (43%)	23 (18.7%)	23 (28%)	303 (51.9%)	112 (62.2%)				
	Median (Q1–Q3)	10 (7–11)	7 (6–10)	9 (7–11)	11 (8–12)	11 (10–12)	.**022**	**<**.**001**	**<**.**001**	**<**.**001**
Maximum number of stools per day	3	22 (18.2%)	28 (22.8%)	13 (15.9%)	92 (15.8%)	25 (13.9%)				
	4–5	69 (57%)	74 (60.2%)	50 (61%)	353 (60.4%)	100 (55.6%)	.603	.083	.992	.466
	6–10	30 (24.8%)	21 (17.1%)	19 (23.2%)	139 (23.8%)	55 (30.6%)				
Days of diarrhea	Median (Q1–Q3)	3 (2–4)	3 (2–3)	3 (2–4)	3 (2–3)	3 (2–4)	.115	.2797	.3293	.259
Vomited	Yes	81 (66.9%)	50 (40.7%)	46 (56.1%)	461 (78.9%)	155 (86.1%)	.**006**	**<**.**001**	**<**.**001**	**<**.**001**
Max number of vomiting episodes per day	1	13 (16%)	14 (28.0%)	9 (19.6%)	43 (9.3%)	17 (11.0%)				
	2–4	56 (69.1%)	34 (68.0%)	31 (67.4%)	324 (70.3%)	99 (63.9%)	.127	**<**.**001**	.074	.115
	≥5	12 (14.8%)	2 (4.0%)	6 (13.0%)	94 (20.4%)	39 (25.2%)				
Days of vomiting	Median (Q1–Q3)	2 (2–3)	2 (1–2)	2 (1–2)	2 (2–3)	2 (1–3)	.735	.**011**	.**002**	.**002**
Axillary temperature	Median (Q1–Q3)	36.8 (36.5–37.3)	36.8 (36.4–37.4)	36.7 (36.5–37.2)	36.9 (36.5–37.4)	36.9 (36.5–37.4)	.184	.394	.081	.058
Dehydration category	None	12 (9.9%)	6 (4.9%)	5 (6.1%)	38 (6.5%)	5 (2.8%)				
	Some	89 (73.6%)	97 (78.9%)	52 (63.4%)	464 (79.5%)	129 (71.7%)	.284	.676	**<**.**001**	.240
	Severe	20 (16.5%)	20 (16.3%)	25 (30.5%)	82 (14.0%)	46 (25.6%)				
Treatment	None	4 (3.3%)	2 (1.6%)	0	18 (3.1%)	0	.521	**<**.**001**	.**017**	**<**.**001**
	Oral rehydration only	102 (84.3%)	117 (95.1%)	76 (92.7%)	469 (80.3%)	134 (74.4%)				
	Intravenous fluids and/or hospitalization	15 (12.4%)	3 (2.4%)	6 (7.3%)	96 (16.4%)	45 (25.0%)				

*P* values were estimated using the *χ*^2^ test or Fisher exact test for categorical variables and the Wilcoxon rank sum test for quantitative variables. Significant values are shown in bold.

To allow for statistical comparisons, moderate-to-severe diarrhea (MSD) cases attributable to more than 1 of adenovirus 40/41, astrovirus, sapovirus, or rotavirus were excluded. Note that there was a small number of MSD cases attributable to 1 of the 4 pathogens listed above who did not have data for all modified Verskiari score measures.

The maximum number of stools per day was similar across the viruses. The proportion of children with vomiting was highest in cases attributed to rotavirus. Vomiting was significantly more common among cases attributed to rotavirus compared with those attributed to the other viruses. However, when compared with rotavirus, the maximum number of vomiting episodes in a day was comparable for adenovirus 40/41 (*P* = .127) and sapovirus (*P* = .074) but significantly lower for astrovirus (*P* < .001).

Children with sapovirus-attributable MSD were only from the Kenya site and were the most likely to be classified in the severe dehydration category. The percentage of cases classified as severe was no longer statistically higher than rotavirus when the dehydration category comparison was restricted to cases from the Kenya site. Regardless of dehydration category, 100% of sapovirus and rotavirus cases in Kenya received oral rehydration, IV fluids, and/or hospitalization (Table 3).

### Seasonality

**Figure 1.**
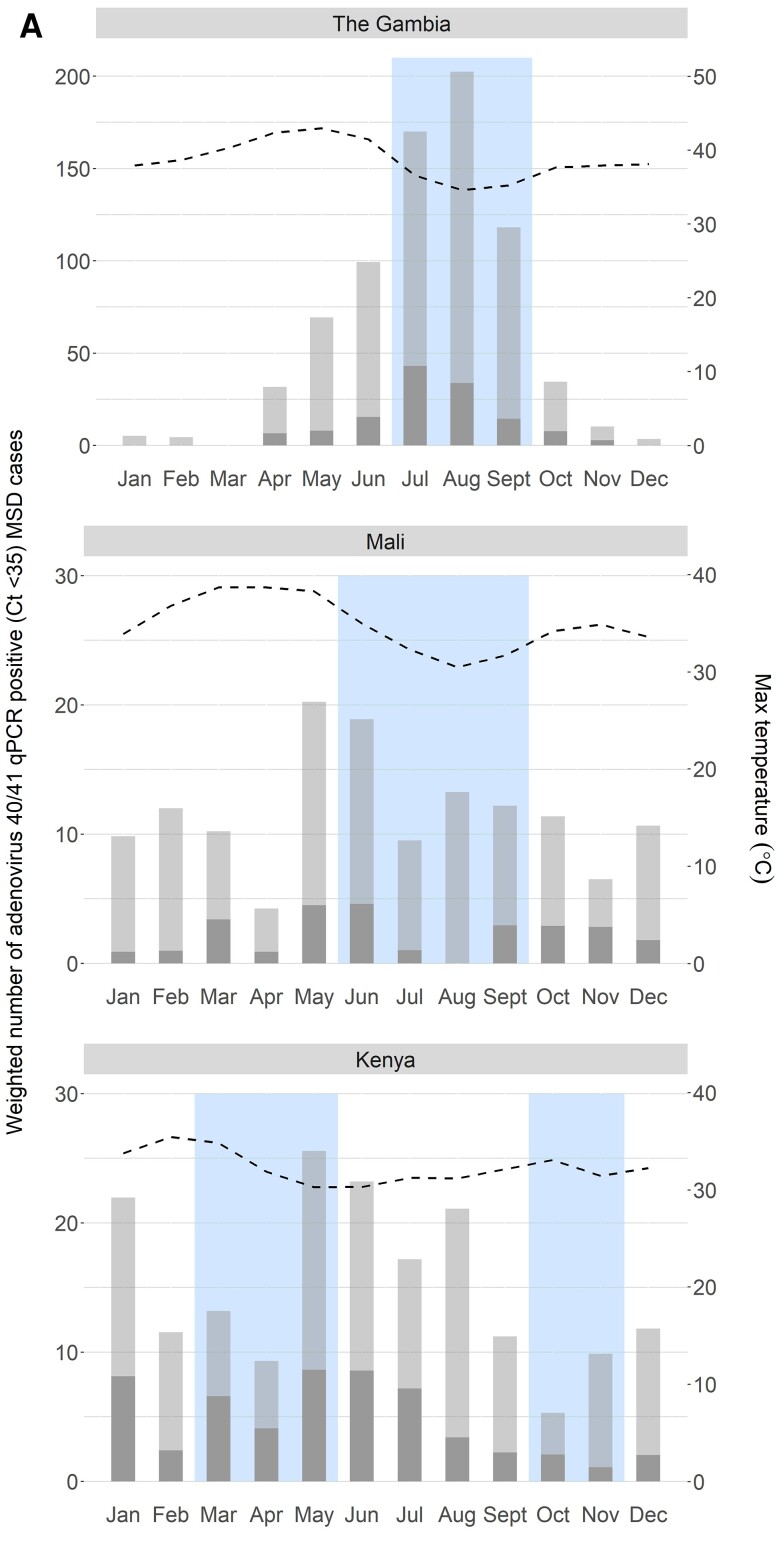

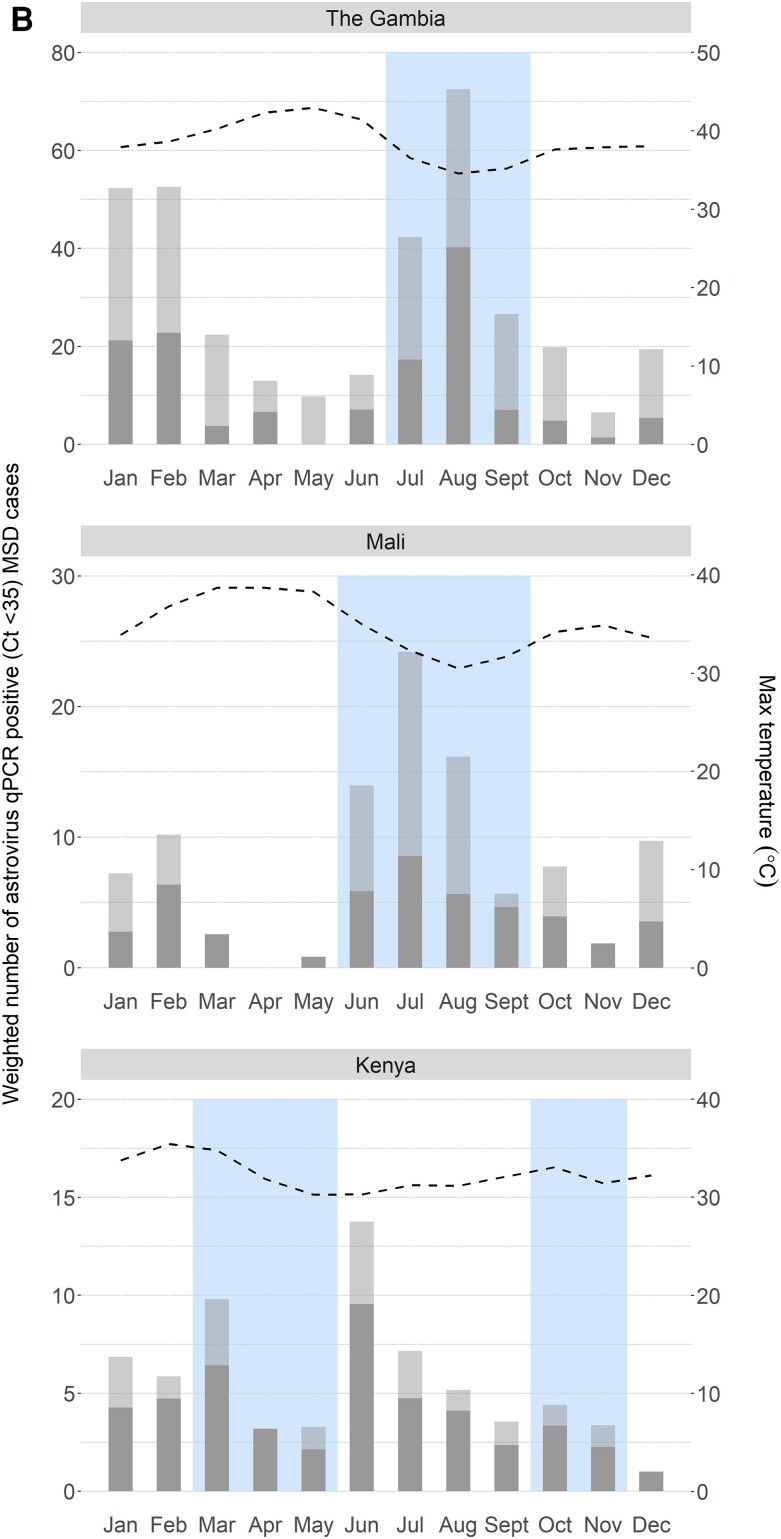

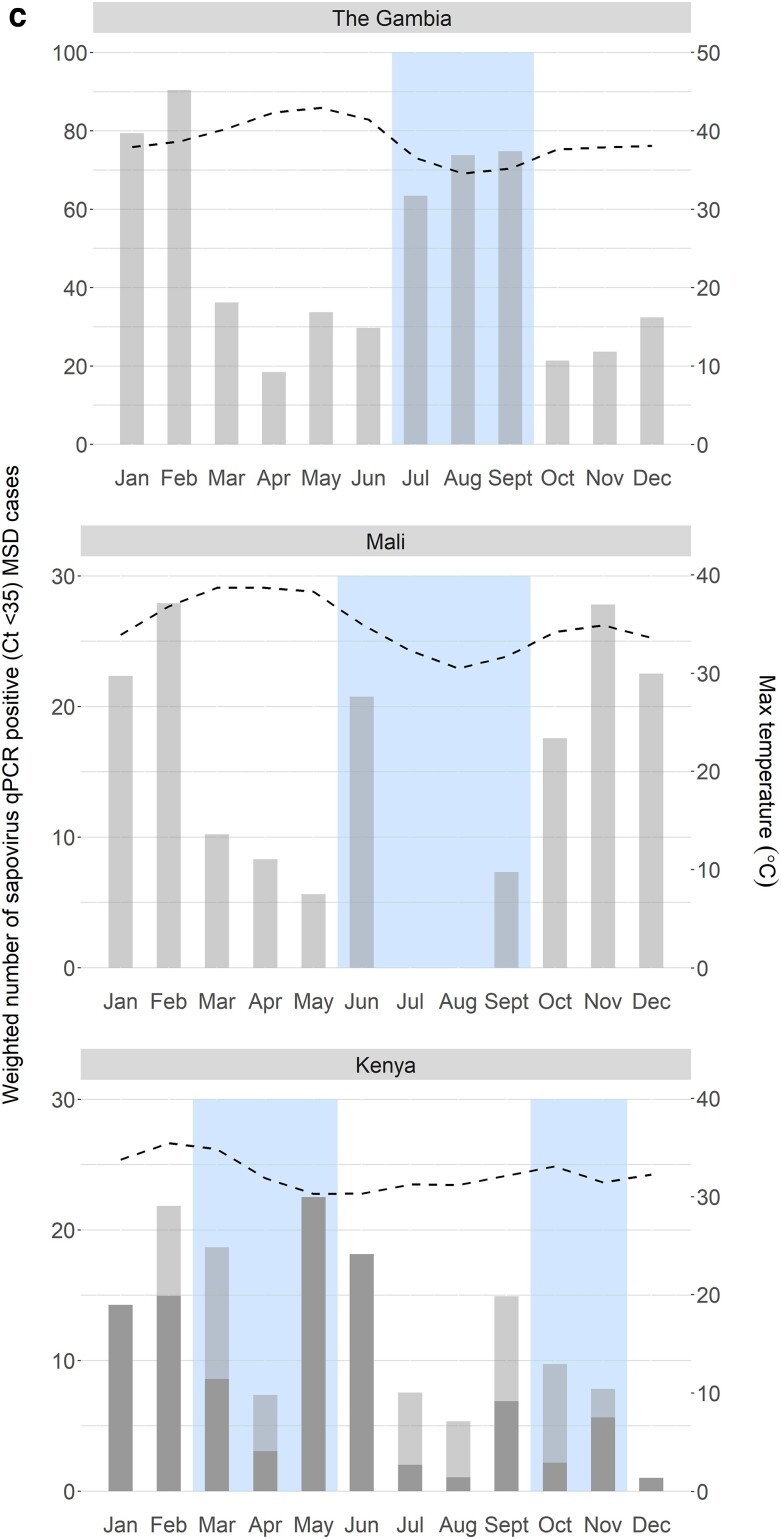

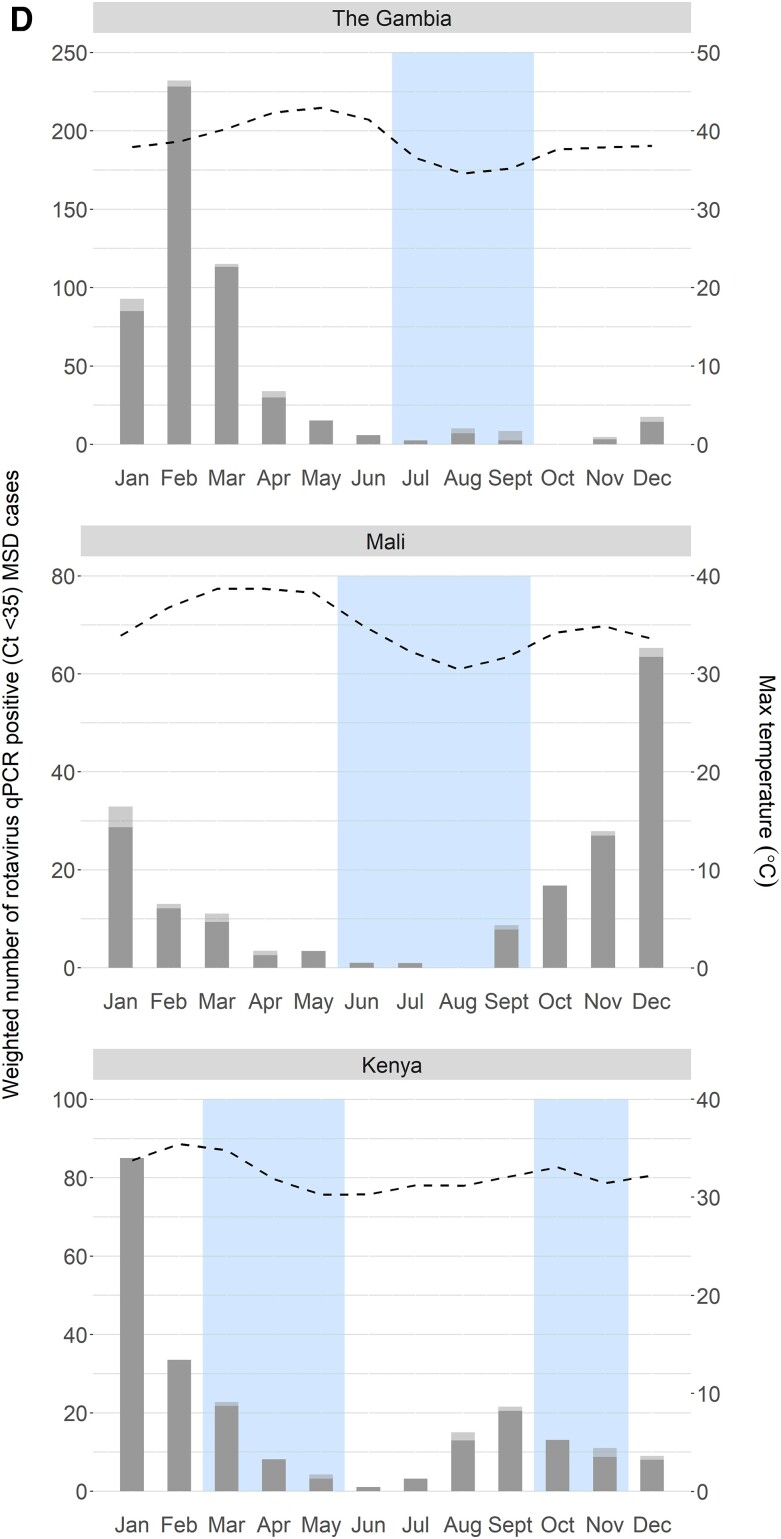
*A,* Seasonality of adenovirus 40/41 at each of the 3 Vaccine Impact on Diarrhea in Africa (VIDA) study sites. The weighted number of qPCR-positive (Ct <35) MSD cases has been divided into nonattributable (light gray) and attributable (dark gray). In addition to the maximum temperature, months with higher-than-average rainfall are indicated by the blue box. *B,* Seasonality of sapovirus at each of the 3 VIDA study sites. The weighted number of qPCR-positive (Ct <35) MSD cases has been divided into nonattributable (light gray) and attributable (dark gray). In addition to the maximum temperature, months with higher-than-average rainfall are indicated by the blue box. *C,* Seasonality of astrovirus at each of the 3 VIDA study sites. The weighted number of qPCR-positive (Ct <35) MSD cases has been divided into nonattributable (light gray) and attributable (dark gray). In addition to the maximum temperature, months with higher-than-average rainfall are indicated by the blue box. *D,* Seasonality of rotavirus at each of the 3 VIDA study sites. The weighted number of qPCR-positive (Ct <35) MSD cases has been divided into nonattributable (light gray) and attributable (dark gray). In addition to the maximum temperature, months with higher-than-average rainfall are indicated by the blue box. Abbreviations: Ct, cycle threshold; MSD, moderate-to-severe diarrhea; qPCR, quantitative polymerase chain reaction.

Figure 1*A–D* summarizes the seasonality of adenovirus 40/41, astrovirus, sapovirus, and rotavirus by site. In The Gambia, we observed an annual peak of adenovirus 40/41-positive MSD cases and a small peak of astrovirus MSD cases peak in August. Both peaks occurred during the rainy season when temperatures were cooler. There was also a smaller astrovirus peak in January during the dry season when temperatures were slightly higher. Rotavirus was predominant outside of the rainy season and peaked in February with no apparent variation by temperature.

In Mali, there were multiple peaks of adenovirus 40/41 cases with almost all in March, May–June, and September–November. The largest peak ended during the rainy season in June, as temperatures began to fall, and the smallest peak lasted several months. There was no clear trend in adenovirus 40/41 cases relative to rainfall with peaks in both the rainy and dry seasons. There were 2 seasonal peaks of astrovirus, both larger than adenovirus 40/41, with the largest peak during the cool, rainy season and the other peak in February when the weather was warmer and drier. The rotavirus season coincided with the dry season.

In Kenya, peaks in adenovirus 40/41 (January) and astrovirus (March) were followed by second peaks in May (adenovirus 40/41) and June (astrovirus). Each peaked during the rainy season and dry season. While there was little variability in temperature, the first adenovirus 40/41 peak occurred during the warmer dry period. There were no clear seasonality trends in sapovirus cases.

## DISCUSSION

MSD continues to negatively impact young children in Africa. Among the 4 viral pathogens included in this analysis, rotavirus is the most common cause of MSD, accounting for 12.6% of all cases. In addition, while less common than rotavirus, adenovirus 40/41, astrovirus, and sapovirus are associated with MSD among young children in Africa, cumulatively accounting for 6.5% of MSD cases. The significance of the association varied by site and age [Kotloff et al., in preparartion]. Given the importance of norovirus in the post-rotavirus vaccine setting and the late stage development of several norovirus vaccines, we report on norovirus infections in a separate article [8]. Researchers found that the norovirus prevalence in cases and controls and the AFe were higher than seen for adenovirus, astrovirus, or sapovirus. As measured by the mVS and percent with severe dehydration, norovirus severity was similar to adenovirus 40/41 infections.

Comparisons of our findings to other etiologic studies of diarrhea must take into consideration the populations and methodologic differences, including the outcome definitions. Whereas GEMS and VIDA found that rotavirus, *Cryptosporidium* spp., and *Shigella* spp. were the most common etiologies of MSD [4,12; Kotloff et al., in preparartion], viruses caused more diarrhea than bacteria and parasites in the Etiology, Risk Factors and Interactions of Enteric Infections and Malnutrition and the Consequences for Child Health and Development (MAL-ED) study, with sapovirus and adenovirus 40/41 as major etiologies in children aged <2 years [24]. Like GEMS and VIDA, MAL-ED measured the proportion of diarrheal disease attributable to a diverse panel of enteropathogens, adjusting for asymptomatic infection in controls. However, GEMS and VIDA aimed to study more severe illness and therefore enrolled episodes that were medically attended in high-mortality, low-resource settings. By design, MAL-ED, a community-based newborn cohort study, captured milder episodes, 75% of which did not seek medical care and only 10% met criteria for MSD. The milder illnesses captured in MAL-ED were seen in the context of a higher prevalence of viral agents. As seen in this analysis, adenovirus 40/41, astrovirus, and sapovirus were common in controls as well as MSD cases, resulting in the lower attributable infections reported herein.

Despite the modest AFe of 2.7%, the impact of adenovirus 40/41 in this analysis is notable with severity being comparable to that of rotavirus MSD by most measures. Likewise, among children aged <1 year, severity of disease measured by mVS was higher for rotavirus and adenovirus 40/41 compared with sapovirus and astrovirus. Adenovirus 40/41 as a cause of MSD, including severe dehydrating diarrhea, was reported from a cohort of hospitalized children in Bangladesh [40], where it was the second leading cause of severe diarrhea in the youngest children. Of note, rotavirus vaccines have not yet been introduced in Bangladesh. That adenovirus causes severe diarrhea is an important finding for future investments in preventive measures, as averting severe clinical outcomes is the highest priority.

While 67 human adenovirus serotypes have been identified to date, adenovirus 40/41 is the most consistently associated with diarrhea and acute gastroenteritis in children [41]. There are, however, other human adenoviruses that cause MSD, but these are uncommon [41]. In VIDA, the qPCR only detected adenovirus 40/41 and might have missed non-40/41 adenoviruses. For example, in GEMS-1A, non-40/41 adenoviruses that caused diarrhea were detected using enzyme immunoassays in Bangladesh and Mali [12].

The frequency of detection of sapovirus is variable across geographic locations and age groups. In a recent review, sapovirus was detected in 1%–15% of children aged <5 years who presented to medical facilities with diarrhea [24, 42]. In MAL-ED, sapovirus detection was higher in younger children and in lower-resource settings [24]. In this study, sapovirus was detected in at least 11% of both cases and controls, and we only identified sapovirus as an etiologic agent of MSD in Kenya. When restricted to the Kenya site, rotavirus and sapovirus MSD cases categorized in the severe dehydration category were high and comparable at 25.6% and 30.5%, respectively. Importantly, administration of treatment was excellent at the Kenya site, with 100% of children with sapovirus or rotavirus receiving rehydration treatment regardless of dehydration category.

Compared to adenovirus 40/41 and sapovirus, astrovirus was detected in fewer cases and controls in this study. However, astrovirus was associated with MSD, with an etiologic fraction of 2.9%. Astroviruses have been strongly associated with diarrhea among young children in several studies, including among children aged <6 months [43]. Astrovirus infection was generally less severe than rotavirus infection, as determined by both mVS and the number of children hospitalized and/or receiving IV treatment for their diarrhea.

The seasonality of virus circulation varied by pathogen and location. The pattern seen in The Gambia for adenovirus 40/41 and astrovirus, favoring the cool, rainy season, is consistent with previous work [44] and possibly explained by spending more time inside due to the cooler temperatures and/or precipitation. Rotavirus peaked during the dry season in Mali and The Gambia, also corroborating seasonal trends seen elsewhere in Africa [45]. Seasonality was not pronounced for any virus in Kenya.

Our study has limitations that need to be considered when interpreting the results. The detection of these viruses is heterogenous across geographies and populations, which may limit the generalizability of our findings. While there is no clear or consistent way to handle coinfections in children with diarrhea, coinfections with 2 or more of the viruses studied herein were uncommon [46]. Finally, while the mVS has been used in assessing rotavirus severity in low-resource settings [7, 38], it has not been validated as a measure of severity for the other viral pathogens.

## CONCLUSIONS

Rotavirus continues to be a frequent cause of MSD, even after rotavirus vaccine introduction, emphasizing the need to optimize rotavirus vaccine coverage while continuing to develop second-generation rotavirus vaccines. Further, with the use of sensitive molecular microbiological diagnostics, adenovirus 40/41, astrovirus, and sapovirus are increasingly recognized as diarrheal pathogens, and a better understanding of risk factors for acquiring these viral infections is needed. Dehydration was common in children who presented with MSD at these sites, regardless of viral etiology, further emphasizing the importance of adequate rehydration in all cases of MSD.

## Supplementary Data

Supplementary materials are available at *Clinical Infectious Diseases* online. Consisting of data provided by the authors to benefit the reader, the posted materials are not copyedited and are the sole responsibility of the authors, so questions or comments should be addressed to the corresponding author.

## Supplementary Material

ciad060_Supplementary_Data

## Notes

***Acknowledgments.*** The authors express their deep gratitude to the families who participated in these studies, the clinical and field staff for their exceptional hard work and dedication, and the physicians, administrators, and health officials at every site who generously provided facilities and support for the conduct of the study. They are grateful to Catherine Johnson, Chris Focht, and Nora Watson at the Emmes Company, LLC, for expert data management and reporting. Special thanks go to Carl Kirkwood, Duncan Steele, and Anita Zaidi at the Bill & Melinda Gates Foundation for helpful oversight and to the following members of the International Scientific Advisory Committee for providing insightful comments and guidance: Janet Wittes (chair), George Armah, John Clemens, Christopher Duggan, Stephane Helleringer, Ali Mokdad, James Nataro, and Halvor Sommerfelt. They thank Professor Larry Magder for his statistical assistance.

***Supplement Sponsorship*.** This supplement was sponsored by the Bill & Melinda Gates Foundation. This study is based on research funded in part by grants from the Bill & Melinda Gates Foundation (OPP1111236/OPP1116751).

***Financial support*.** This work was supported by grants from the Bill & Melinda Gates Foundation (OPP1111236 and OPP1116751).
