# Supplementary material for: Prevalence, Clinical Severity, and Seasonality of Adenovirus 40/41, Astrovirus, Sapovirus, and Rotavirus Among Young Children With Moderate-to-Severe Diarrhea: Results From the Vaccine Impact on Diarrhea in Africa (VIDA) Study
Source: Clin Infect Dis. 2023 Apr 19;76(Suppl 1):S123–31. doi: 10.1093/cid/ciad060 (PMC10116545; doi:10.1093/cid/ciad060)
Supplement: ciad060_Supplementary_Data [file ciad060_supplementary_data.docx]

**Supplementary Appendix**

**Prevalence, clinical severity, and seasonality of adenovirus 40/41, astrovirus, and sapovirus among young children with moderate-to-severe diarrhea: Results from the Vaccine Impact on Diarrhea in Africa (VIDA) study**

Authors

Adama M Keita, Sanogo Doh, Samba O Sow, Helen Powell, Richard Omore, M Jahangir Hossain, Billy Ogwel, John Benjamin Ochieng, Joquina Chiquita M Jones, Syed MA Zaman, Alex O Awuor, Jane Juma, Dilruba Nasrin, Jie Liu, Awa Traoré, Uma Onwuchekwa, Henry Badji, Golam Sarwar, Martin Antonio, Eric Houpt, Sharon M Tennant, Irene N Kasumba, Leslie P Jamka, Anna Roose, James Platts-Mills, Jacqueline E Tate, Umesh D Parashar, Kathleen M Neuzil, and Karen L Kotloff

| **Supplementary Table 1. Comparison of Vesikari Score and the modified Vesikari Score (mVS) used in VIDA** | | | | | | |
| --- | --- | --- | --- | --- | --- | --- |
| **Parameter** | **Score** | | | | | **Comment** |
|  | **Vesikari** | | | | |  |
|  | **1** | **2** | | | **3** |  |
| Diarrhea |  |  | | |  |  |
| Max. no. stools/day | 1-3 | 4-5 | | | >6 |  |
| Duration (days) | 1-4 | 5 | | | >6 |  |
| Vomiting |  |  | | |  |  |
| Max. no. emesis/day | 1 | 2-4 | | | >5 |  |
| Duration (days) | 1 | 2 | | | >3 |  |
| Temperature (°C) | 37.1-38.4 | 38.5-38.9 | | | >39.0 |  |
| Dehydration | N/A | 1-5% or some | | | >6% or severe | . |
| Treatment | Rehydration | Hospitalization | | | N/A | Participants who are “hospitalized” for at least 24 hours OR who receive IV therapy are considered “hospitalized” and receive a corresponding score of 2 points for this parameter |
|  | **VIDA Modified Vesikari Score (mVS)** | | | | |  |
| Diarrhea |  | |  |  | |  |
| Max. no. stools/day | 3 | | 4-5 | >6 | | 1-2 days not permissible; definition of diarrhea requires >3 stools/day |
| Duration (days) | 1-4 | | 5 | 6-7 | | Cannot exceed 7; enrolment criteria requires <7 days |
| Vomiting |  | |  |  | |  |
| Max. no. emesis/day | 1 | | 2-4 | >5 | |  |
| Duration (days) | 1 | | 2 | >3 | | May be truncated because of diarrhea duration enrolment criterion |
| Temperature (°C) | 37.1-38.4 | | 38.5-38.9 | >39.0 | | No change |
| Dehydration | N/A | | some | severe | | No change |
| Treatment | Rehydration | | Hospitalization/IV | N/A | | No change |
|  |  | |  |  | |  |

Supplementary Table 2: Median (Q1, Q3) modified Vesikari score by virus and age group

|  | Adenovirus | Astrovirus | Sapovirus | Rotavirus |
| --- | --- | --- | --- | --- |
| 0-11 months | N= 70  10.5 (7.0, 11.75) | N=33  7.0 (6.0,10.0) | N=23  9.0 (8.5, 11.0) | N=253  11.0 (8.0, 12.0) |
| 12-23 months | N=39  10.0 (7.0, 11.0) | N=71  7.5 (6.0, 10.0) | N=33  10.0 (7.0, 11.0) | N=219  11.0 (8.0, 12.0) |
| 24-59 months | N=12  8.0 (6.75, 11.25) | N=19  9.0 (6.0, 10.5) | N=26  8.5 (6.0, 10.0) | N=112  10.0 (8.0, 12.0) |
